# Supplementary material for: Clinical outcomes associated with schistosome infection and alcohol use: A systematic scoping review
Source: PLoS Negl Trop Dis. 2026 May 27;20(5):e0014371. doi: 10.1371/journal.pntd.0014371 (PMC13215488; doi:10.1371/journal.pntd.0014371)
Supplement: Attachment — Submitted filename: Reviewer Comments_PLoS NTD Schisto and Alcohol.docx [file pntd.0014371.s005.docx]

Systematic scoping review exploring the link between schistosomiasis and alcohol use co-systematic scoping review examining the intersection of schistosomiasis and alcohol use as co-occurring risk factors for liver and other systemic pathologies. The topic is timely, particularly given the growing recognition of dual burdens of infectious and non-communicable diseases in low- and middle-income countries. The paper is clearly written, follows PRISMA-ScR guidelines, and shows commendable attention to methodological rigor.

However, despite its clear presentation and adherence to scoping review standards, the conceptual and analytical contributions remain modest, and the narrative synthesis could be strengthened to yield deeper insights beyond descriptive mapping. The various outcomes could also be better organized in the text and tables. With moderate revision, the paper could become suitable for publication. Thank you for allowing me to review this paper.

**Major Comments**

**Novelty and Conceptual Framing**

- **Strengths:**
  The study fills an important gap by systematically mapping studies that assess co-occurring schistosome infection and alcohol use, an underexplored area.
- **Concerns:**
  The introduction frames a biologically plausible synergism between the two exposures, but this is not fully developed or tested in the synthesis.
  **Suggestion:** Expand the conceptual model — perhaps visually — showing hypothesized pathways (immune modulation, fibrosis acceleration, hepcidin regulation). This could tie together the scattered discussion of “synergistic” versus “antagonistic” findings and highlight the review’s conceptual contribution.

**Scope and Research Question Definition**

- The stated objective (“to assess existing evidence on the combined effect of schistosomiasis and alcohol use on clinical outcomes”) is clear but broad.
  Yet, the review merges *different outcome domains* (hepatic, iron-related, infectious comorbidities) without sufficient justification for grouping them under one analytical umbrella.
  **Suggestion:** Reorganize results around *pathophysiological mechanisms* or *disease domains* rather than study design or country. This would help readers see coherent patterns rather than a list of heterogeneous results. It would also be nice to see which studies were longitudinal and which were cross-sectional, and whether the studies examined treatment of schistosome infection and/or EtOH use and how that impacted outcomes. Additionally, the quantification of alcohol use and the definition of schistosome infection were too variable. I am not sure, after reading through this manuscript and trying to understand all the outcomes, whether the authors can conclude that co-occurring schistosomiasis and alcohol use is associated with more severe liver disease.

**Methodological Rigor and Search Strategy**

- The use of multiple databases (PubMed, Embase, Web of Science, Global Index Medicus, Global Health) is exemplary.
- However, the search terms (“(Schistosom* OR Bilharzia* OR snail* fever) AND (alcohol OR drink*)”) may have been too narrow, possibly excluding relevant work using terms like “ethanol,” “substance use,” or “alcohol consumption disorder.”
  Suggestion: Discuss limitations of the search string explicitly and consider including a sensitivity analysis or supplementary search strategy.

**Data Synthesis and Interpretation**

- The results section is thorough, but the synthesis stops short of meta-inference. It mostly catalogues study characteristics (Tables 1–2) without integrating patterns.
  For example, no attempt is made to map which outcomes are likely to be confounded by age, sex, or socioeconomic status, though the authors note confounding qualitatively.
  Suggestion:
  - Include a summary figure or matrix showing the direction of effects (positive/negative/null) by outcome and exposure.
  - Consider a “vote-counting” or narrative meta-summary that shows trends across hepatic vs. hematologic outcomes.

**Discussion and Interpretation of Mechanisms**

- The discussion (pages 22–24) does an admirable job outlining potential biological pathways, but it should better connect animal findings with human data.
  Currently, murine studies are cited without addressing translational limits (dose, exposure duration, species differences).

Suggestion: Add a paragraph critically comparing animal and human data to strengthen mechanistic plausibility while maintaining caution.

**Limitations**

- Scoping reviews sometimes also undergo quality assessment of the full texts to ensure the studies included are rigorous. Was this done or considered? Additionally, full-text reviews are typically conducted by two people.
