# Supplementary material for: Clinical outcomes associated with schistosome infection and alcohol use: A systematic scoping review
Source: PLoS Negl Trop Dis. 2026 May 27;20(5):e0014371. doi: 10.1371/journal.pntd.0014371 (PMC13215488; doi:10.1371/journal.pntd.0014371)
Supplement: Attachment — Submitted filename: Response to reviewers for scoping review 20260113.docx [file pntd.0014371.s007.docx]

Response to reviewers for manuscript: “Clinical outcomes associated with schistosome infection and alcohol use: a systematic scoping review”.

EDITOR’S COMMENTS

Comment 1

- Please upload all main figures as separate Figure files in .tif or .eps format. For more information about how to convert and format your figure files please see our guidelines:
   https://journals.plos.org/plosntds/s/figures

*Response:*

There is one main figure, and is has been uploaded in .tif format.

Comment 2

- Tables should not be uploaded as individual files. Please remove these files as they should only be included in the manuscript file as editable, cell-based objects. For more information about how to format tables, see our guidelines:
   <https://journals.plos.org/plosntds/s/tables>

*Response:*

Tables have only been included in the manuscript file as editable cell-based objects and have not been uploaded as separate documents.

Comment 3

- Please amend your detailed Financial Disclosure statement. This is published with the article. It must therefore be completed in full sentences and contain the exact wording you wish to be published. State what role the funders took in the study. If the funders had no role in your study, please state: "The funders had no role in study design, data collection and analysis, decision to publish, or preparation of the manuscript."

*Response:*

The statement indicated above has been added to the Financial Disclosure section.

Comment 4

- Please ensure that the funders and grant numbers match between the Financial Disclosure field and the Funding Information tab in your submission form. Note that the funders must be provided in the same order in both places as well. Currently, the order of the funders is different in both places.

*Response:*

We apologise for this error, and have provided the order of the funders so that it is the same in both places.

Comment 5

- As required by our policy on Data Availability, please ensure your manuscript or supplementary information includes the following:
   - A numbered table of all studies identified in the literature search, including those that were excluded from the analyses.

*Response:*

This has been included as a supplementary file (S3), and has been indicated in the main text (Results, paragraph 1).

Comment 6

- For every excluded study, the table should list the reason(s) for exclusion. If any of the included studies are unpublished, include a link (URL) to the primary source or detailed information about how the content can be accessed.

*Response:*

The reasons for exclusion have been included in the same table in Supplementary File S3.

Comment 7

- A table of all data extracted from the primary research sources for the systematic review and/or meta-analysis should be included. The table must include the following information for each study:
   - Name of data extractors and date of data extraction
   - Confirmation that the study was eligible to be included in the review.
   - All data extracted from each study for the reported systematic review and/or meta-analysis that would be needed to replicate your analyses.

*Response:*

This has been included as Supplementary File 2, and has been indicated in the main text (Methods, paragraph 4).

Comment 8

- If data or supporting information were obtained from another source (e.g. correspondence with the author of the original research article), please provide the source of data and dates on which the data/information were obtained by your research group.

*Response:*

Authors were contacted for missing information during the extraction phase of the review. Of the 14 authors who were contacted, three authors responded to provide missing data (all in June 2025).

Comment 9

- If applicable for your analysis, a table showing the completed risk of bias and quality/certainty assessments for each study or outcome. Please ensure this is provided for each domain or parameter assessed. For example, if you used the Cochrane risk-of-bias tool for randomized trials, provide answers to each of the signalling questions for each study. If you used GRADE to assess certainty of evidence, provide judgements about each of the quality of evidence factor. This should be provided for each outcome.

*Response:*

A formal risk of bias assessment is not compulsory for a scoping review (Pollock et al., 2023), and so a table has not been included.

Comment 10

- An explanation of how missing data were handled. This information can be included in the main text, supplementary information, or relevant data repository. Please note that providing these underlying data is a requirement for publication in this journal.

*Response:*

Missing data was initially indicated by entering “unclear” into the data extraction table. Where a study had any data points marked as unclear, two attempts were made to contact the corresponding author of the study via email.

References

Pollock, D., Peters, M. D. J., Khalil, H., McInerney, P., Alexander, L., Tricco, A. C., Evans, C., de Moraes, É. B., Godfrey, C. M., Pieper, D., Saran, A., Stern, C., & Munn, Z. (2023). Recommendations for the extraction, analysis, and presentation of results in scoping reviews. *JBI Evidence Synthesis*, *21*(3), 520–532. https://doi.org/10.11124/JBIES-22-00123

REVIEWERS’ COMMENTS

Reviewer 1

Comment 1

- Systematic scoping review exploring the link between schistosomiasis and alcohol use as co-occurring risk factors for liver and other systemic pathologies. The topic is timely, particularly given the growing recognition of dual burdens of infectious and non-communicable diseases in low- and middle-income countries. The paper is clearly written, follows PRISMA-ScR guidelines, and shows commendable attention to methodological rigor. However, despite its clear presentation and adherence to scoping review standards, the conceptual and analytical contributions remain modest, and the narrative synthesis could be strengthened to yield deeper insights beyond descriptive mapping. The various outcomes could also be better organized in the text and tables. With moderate revision, the paper could become suitable for publication. Thank you for allowing me to review this paper.

*Response:*

We thank the reviewer for taking the time to read this scoping review and for their comments, which have greatly improved the manuscript.

Comment 2

- The introduction frames a biologically plausible synergism between the two exposures, but this is not fully developed or tested in the synthesis. Suggestion: Expand the conceptual model — perhaps visually — showing hypothesized pathways (immune modulation, fibrosis acceleration, hepcidin regulation). This could tie together the scattered discussion of “synergistic” versus “antagonistic” findings and highlight the review’s conceptual contribution.

*Response:*

We thank the reviewer for these suggestions, and agree that a clearer conceptual framework would strengthen the synthesis. In response, we have expanded the “Disease outcomes and hypothesised pathways” subsection of the Discussion (paragraphs 2-5) to organise evidence around plausible biological mechanisms that consider both synergistic and antagonistic effects. We have refined the language used throughout the discussion and softened the claim of direct synergy to better reflect the complexity of the evidence.

Comment 3

- The stated objective (“to assess existing evidence on the combined effect of schistosomiasis and alcohol use on clinical outcomes”) is clear but broad. Yet, the review merges *different outcome domains* (hepatic, iron-related, infectious comorbidities) without sufficient justification for grouping them under one analytical umbrella. Suggestion: Reorganize results around *pathophysiological mechanisms* or *disease domains* rather than study design or country. This would help readers see coherent patterns rather than a list of heterogeneous results. It would also be nice to see which studies were longitudinal and which were cross-sectional, and whether the studies examined treatment of schistosome infection and/or EtOH use and how that impacted outcomes. Additionally, the quantification of alcohol use and the definition of schistosome infection were too variable. I am not sure, after reading through this manuscript and trying to understand all the outcomes, whether the authors can conclude that co-occurring schistosomiasis and alcohol use is associated with more severe liver disease.

*Response:*

We thank the reviewer for these comments and suggestions. The “Study characteristics” subsection of the Results was included to follow standard reporting procedures for scoping reviews (Tricco et al., 2018). We have, however, reorganised the subsection titled “Associations of alcohol use and schistosome infection to disease outcomes” to group findings by outcome domain, as suggested. We have also added the study design for each included study to Table 2. Only one study reported on schistosomiasis treatment, and this has been referenced in the text in the same subsection: “A cohort study (Li et al., 2000), which evaluated treatment, found that moderate alcohol consumption was associated with lower odds of PPF improvement following praziquantel, suggesting a potential exacerbating effect.”

We further acknowledge the substantial variability in how alcohol use and schistosome infection exposure were defined and measured across studies, and we have accordingly softened our conclusions. Rather than asserting a synergistic relationship, we now conclude that the available evidence suggests potential synergistic and antagonistic pathways, while emphasising the limitations imposed by the study heterogeneity and predominance of cross-sectional designs.

Comment 4

- The use of multiple databases (PubMed, Embase, Web of Science, Global Index Medicus, Global Health) is exemplary. However, the search terms (“(Schistosom* OR Bilharzia* OR snail* fever) AND (alcohol OR drink*)”) may have been too narrow, possibly excluding relevant work using terms like “ethanol,” “substance use,” or “alcohol consumption disorder.” Suggestion: Discuss limitations of the search string explicitly and consider including a sensitivity analysis or supplementary search strategy.

*Response:*

Thank you for highlighting where clarification was needed. We only provided the general search string in the text. Detailed search terms are provided in Supplementary File S1. The search strategy was developed through an iterative process, involving exploratory searches and consultation with librarians at the Bodleian University of Oxford library. In each database, the search string was supplemented with controlled vocabulary (e.g. MeSH), which incorporated related words including ethanol, substance use, or alcohol use disorder. Additionally, three papers were used to validate both the search string and data extraction table(Kavishe et al., 2021; Malenganisho et al., 2008; Ocama et al., 2017).

Comment 5

- The results section is thorough, but the synthesis stops short of meta-inference. It mostly catalogues study characteristics (Tables 1–2) without integrating patterns. For example, no attempt is made to map which outcomes are likely to be confounded by age, sex, or socioeconomic status, though the authors note confounding qualitatively. Suggestion:
  - Include a summary figure or matrix showing the direction of effects (positive/negative/null) by outcome and exposure.
  - Consider a “vote-counting” or narrative meta-summary that shows trends across hepatic vs. hematologic outcomes.

*Response:*

We thank the reviewer for this helpful suggestion to enhance the synthesis. In response we have created Table 3, included in the Results section, which presents the suggested summary matrix. It synthesises data across studies to show the direction of effect for key confounders (age, sex, HIV and smoking status) on the two most common outcomes in the review – periportal fibrosis and iron deficiency. It has been stratified by schistosome infection diagnostic method and adjustment status to allow for an assessment of which outcomes are likely to be confounded. We have removed the long text on confounding so that emphasis is placed on the table and its synthesis.

Comment 6

- The discussion (pages 22–24) does an admirable job outlining potential biological pathways, but it should better connect animal findings with human data. Currently, murine studies are cited without addressing translational limits (dose, exposure duration, species differences). Suggestion: Add a paragraph critically comparing animal and human data to strengthen mechanistic plausibility while maintaining caution.

*Response:*

We thank the reviewer for this suggestion. To explicitly acknowledge the translational limits of murine studies, we have added the following to the manuscript: “Although both synergistic and antagonistic relationships have support from murine studies, their translational relevance to human disease is limited. Firstly, experimental models typically administer the exposures in close temporal proximity in adult animals (Brandão-Bezerra et al., 2022; Orrego et al., 1981; Rosa et al., 2024), which is not an accurate reflection of exposure in humans – schistosome infection typically begins in early childhood, before alcohol use is initiated later in life. Furthermore, the controlled dose of each exposure in mice does not reflect the heterogeneity in timing and dose that occurs in human populations, for example when considering behaviours such as binge drinking. Therefore, murine models alone would not be able to resolve which kind of interaction prevails for humans. Instead, prospective human studies over much longer periods of time, such as those studies underway in SchistoTrack (Puthur et al., 2025), are needed.” (Discussion, paragraph 5)

Comment 7

- Scoping reviews sometimes also undergo quality assessment of the full texts to ensure the studies included are rigorous. Was this done or considered? Additionally, full-text reviews are typically conducted by two people.

*Response:*

We thank the reviewer for this comment. Formal quality appraisal is not mandatory for scoping reviews (Tricco et al., 2018), as their aim is to map the existing literature rather than to calculate effect estimates. Therefore, we did not conduct a formal quality assessment of the included studies. Study selection was conducted by a single reviewer, with a random 10% of full text articles screened by a second review to ensure consistency. Any disagreements were resolved through discussion. This approach is consistent with scoping review methodology guidance from the Joanna Briggs Institute (Pollock et al., 2023).

References

Brandão-Bezerra, L., Rosa, A. A. da, Oliveira, R. M. F. de, Neves, R. H., Corrêa, C. L., & Machado-Silva, J. R. (2022). Impact of acute schistosomiasis mansoni and long-term ethanol intake on mouse liver pathology. *Experimental Parasitology*, *242*. https://doi.org/10.1016/J.EXPPARA.2022.108388

Kavishe, B. B., Kweka, B. V., Nitsch, D., PrayGod, G., Jeremiah, K., Faurholt-Jepsen, D., Filteau, S., Olsen, M. F., Kitilya, B. W., Krogh-Madsen, R., Friis, H., & Peck, R. (2021). Risk factors for impaired renal function in HIV-infected and HIV-uninfected adults: cross-sectional study in North-Western Tanzania. *BMC Nephrology*, *22*(1), 1–12. https://doi.org/10.1186/S12882-021-02563-Z/TABLES/4

Malenganisho, W. L. M., Magnussen, P., Friis, H., Siza, J., Kaatano, G., Temu, M., & Vennervald, B. J. (2008). Schistosoma mansoni morbidity among adults in two villages along Lake Victoria shores in Mwanza District, Tanzania. Transactions of the Royal Society of Tropical Medicine and Hygiene, 102(6), 532–541. https://doi.org/10.1016/J.TRSTMH.2008.03.006/2/102-6-532-FIG005.GIF

Ocama, P., Opio, K. C., Seremba, E., Ajal, P., Apica, B. S., & Aginya, E. O. (2017). The burden, pattern and factors that contribute to periportal fibrosis in HIV-infected patients in an S. mansoni endemic rural Uganda. African Health Sciences, 17(2), 301–307. https://doi.org/10.4314/AHS.V17I2.2

Orrego, H., Israel, Y., Crossley, I. R., Mahmoud, A. A. F., Peters, P. A., Varghese, G., & Wanless, I. R. (1981). Effect of chronic alcohol intake on hepatic fibrosis and granulomas in marine schistosomiasis mansoni. Hepatology, 1(5), 416–418. https://doi.org/10.1002/HEP.1840010508

Pollock, D., Peters, M. D. J., Khalil, H., McInerney, P., Alexander, L., Tricco, A. C., Evans, C., de Moraes, É. B., Godfrey, C. M., Pieper, D., Saran, A., Stern, C., & Munn, Z. (2023). Recommendations for the extraction, analysis, and presentation of results in scoping reviews. JBI Evidence Synthesis, 21(3), 520–532. https://doi.org/10.11124/JBIES-22-00123

Puthur, C., Nabatte, B., Tinkitina, B., Kabatereine, N. B., Chami, G. F., Goylette, D., & Chami, F. (2025). Design and maintenance of community-based cohorts in sub-Saharan Africa: a longitudinal evaluation of participant attrition in SchistoTrack. BMJ Public Health, 3(2), e002151. https://doi.org/10.1136/BMJPH-2024-002151

Rosa, A. A. da, Brandão-Bezerra, L., Corrêa, C. L., Amaral G Da-Silva, S., Rodrigues, L. S., Machado-Silva, J. R., & Neves, R. H. (2024). Changes in splenic tissue and immune response profile of Schistosoma mansoni infected mice submitted to chronic ethanol intake. Experimental Parasitology, 259. https://doi.org/10.1016/J.EXPPARA.2024.108706

Tricco, A. C., Lillie, E., Zarin, W., O’Brien, K. K., Colquhoun, H., Levac, D., Moher, D., Peters, M. D. J., Horsley, T., Weeks, L., Hempel, S., Akl, E. A., Chang, C., McGowan, J., Stewart, L., Hartling, L., Aldcroft, A., Wilson, M. G., Garritty, C., … Straus, S. E. (2018). PRISMA extension for scoping reviews (PRISMA-ScR): Checklist and explanation. Annals of Internal Medicine, 169(7), 467–473. https://doi.org/10.7326/M18-0850/SUPPL_FILE/M18-0850_SUPPLEMENT.PDF

Reviewer 2

Comment 1

- The review by Lyne et al. summarizes the results of several studies to conclude that there is a synergy between alcohol consumption and schistosome infection in terms of liver pathology. However, most of the included studies did not explicitly measure or model joint exposure or effect modification. Therefore, it is critical to note that systematic, correlated classifications of alcohol consumption and schistosome infection in primary studies undermine the reliability of joint exposure categories used to infer synergies for liver disease. Without correction, the observed patterns may reflect measurement error rather than true joint effects. Self-reported underreporting of alcohol consumption and false-negative results in single Kato-Katz samples may together lead to individuals being classified into lower exposure categories for both factors.

*Response:*

We thank the reviewer for highlighting these limitations. We have more clearly acknowledged these limitations in the Limitations subsection of the Discussion by adding: “A further limitation relates to the interpretation of joint effects between alcohol use and schistosome infection. None of the studies reported formally testing interaction terms – instead, alcohol use and schistosome infection were measured independently using exposure classifications that are subject to measurement error. In the context of joint exposure assessment, correlated misclassification, arising from underreporting of alcohol consumption or false-negative schistosome infection classification when single-sample microscopy is used, may have resulted in individuals being misclassified into lower exposure categories for both factors. Consequently, some observed associations may reflect measurement error rather than true joint biological effects. Future studies could mitigate these limitations by using repeated measures of alcohol consumption in a subsample of participants, and by applying more reliable schistosomiasis exposure assessment strategies, such as repeated stool sampling or the use of more sensitive antigen-based diagnostics.” (Discussion, paragraph 9).

Comment 2

- The evidence summarized in the review is predominantly cross-sectional (Table 1), and alcohol consumption was typically measured at a single point in time using heterogeneous, self-reported definitions (Table 2). The discussion explicitly acknowledges the lack of temporal information and the limited ability to assess the sequence and duration of exposures, which is critical for determining whether the combined effects are synergistic over the lifespan. Please comment on that.

*Response:*

We thank the reviewer for highlighting these points. In the Discussion, we have refined the conceptual framing of potential pathways in the “Disease outcomes and hypothesised pathways” subsection (paragraphs 2-5). We now explicitly acknowledge that given the methodological and translational limitations of murine studies, the current human evidence does not support synergism over antagonism. The revised text softens our original argument of synergism over antagonism and presents a more balanced view of the existing evidence.

Comment 3

- The PRISMA flow in Figure 1 and the study-level details in Table 1 and Table 2 support a qualitative synthesis confirming synergistic hepatic effects, but the review does not perform a quantitative meta-analysis or meta-regression to formally estimate and test additive or multiplicative interactions between alcohol consumption and schistosome infection with regard to hepatic outcomes. While individual studies demonstrate strong interactions, there is no comprehensive meta-analysis summarizing these effect sizes. Without pooled effect sizes and interaction tests, the conclusion of synergistic effects is based on narrative patterns and isolated study results that are susceptible to bias and heterogeneity at the study level. A formal meta-analysis with random effects and a meta-regression would quantify the variance between studies and test whether the joint exposure effect exceeds the sum or product of the individual exposure effects. Perhaps the authors can comment on this and supplement the proposed calculations.

*Response:*

We appreciate this suggestion, and understand that a meta-analysis would provide a more quantitative assessment of the interaction effects. However, this review was prospectively registered as a scoping review in on 26 February 2025 (Lyne et al., 2025) as we anticipated substantial heterogeneity in outcome and study designs. For this reason, no meta-analysis was planned or conducted, and we have used a qualitative synthesis approach. However, we have now included a table (Table 3, Results) which summarises key confounders and the reported direction of the effects across studies, which allows for easier comparison of patterns within the literature.

We also acknowledge the reviewer’s concern regarding the interpretation of synergistic effects, and have revised the discussion and conclusions to show a conceptual framework of pathways that could be either synergistic or antagonistic.

References

Lyne, B., Lang, M., Lewington, S., & Chami, G. (2025). Clinical outcomes associated with schistosome infection and alcohol use: a systematic scoping review protocol. https://osf.io/https://osf.io/s6c8t

Reviewer 3

Comment 1

- The hypothesis needs to be stated.

*Response:*

We thank the reviewer for noticing this omission. We have added: “The key overarching hypothesis leading into this review is that the presence of schistosome infection, whether past or current, with alcohol use leads to worse liver outcomes and the effect is primarily synergistic.” (Introduction, paragraph 4).

Comment 2

- 21 papers were selected for the review, authors need to include population numbers.

*Response:*

Thank you for this suggestion. The population of each study has been added to Table 2 in the Results section.

Comment 3

- Figure 1- the authors state that there was 1 additional study from other sources, can they specify the source

*Response:*

We thank the reviewer for this comment. The additional study was a previously published study by the authors’ research group, which was identified through searching the authors’ publication records, rather than through the database searches. The study met all predefined inclusion criteria and was assessed using the same screening procedures as other included studies.

Comment 4

- Figure 1: Under screening for 164 studies excluded: the breakdown of various studies does to add up to 164, if there is overlap this needs correcting

*Response:*

We apologise for this error, and thank the reviewer for noticing it. There is no overlap, and the numbers have been corrected (Figure 1, Results section).

Comment 5

- Table 2: include population numbers and age band where available for the 21 papers in the table

*Response:*

We appreciate this suggestion. Both the population numbers and age bands (where reported) have been added to Table 2 in the Results section.

Comment 6

- The discussion sufficiently addresses disease outcomes, however in Line 180- the authors give examples of chronic, heavy alcohol use among the older age group. None of the studies clearly define chronic or heavy alcohol use, nor do they specify how these categories were established (Anjorin et al., 2024). It would be beneficial if the authors could provide clarification on these definitions.

*Response:*

We thank the reviewer for highlighting this point, and agree that definitions of chronic or heavy alcohol use were inconsistently reported across the included studies. As detailed in the Results section (paragraph 5), most studies defined alcohol use using binary or author-defined categories, with substantial variation in thresholds, reference groups, and time frames for alcohol consumption. The examples of chronic of heavy alcohol use referred to in this part of the Discussion reflect the terminology used by the original study authors. We have clarified this in the Discussion (paragraph 2) with: “However, there is a need for future studies to more clearly define chronic or heavy alcohol use.”

Comment 7

- The methods used to diagnosis vary between majority of papers using microscopy, two antigen-based test and one biopsy how does this impact on study outcomes? any limitations?

*Response:*

We thank the reviewer for raising this important point. The heterogeneity in schistosome infection diagnostic methods is a significant source of potential misclassification bias that could impact the observed associations. To address this, we have stratified Table 3 in the Results section by schistosome infection diagnostic method to allow for a visual comparison of how the direction of common confounder associations differ between studies that use different diagnostic methods. The table shows that patterns are difficult to assess for antigen tests due to only one study on periportal fibrosis using a CCA urine text. We have also expanded the Limitations section of the Discussion to acknowledge this issue, by adding: “In the context of joint exposure assessment, correlated misclassification, arising from underreporting of alcohol consumption or false-negative schistosome infection classification when single-sample microscopy is used, may have resulted in individuals being misclassified into lower exposure categories for both factors. Consequently, some observed associations may reflect measurement error rather than true joint biological effects. Future studies could mitigate these limitations by using repeated measures of alcohol consumption in a subsample of participants, and by applying more reliable schistosomiasis exposure assessment strategies, such as repeated stool sampling or the use of more sensitive antigen-based diagnostics.” (Discussion, paragraph 9).

Comment 8

- The authors have undertaken a systematic scoping review to address the how schistosome infection and alcohol use influence health outcomes specifically: clinical outcomes, biological and temporal pathways. The study is a relatively new area with paucity of data. The authors were able to identify 21 publications selected over the past 32 years. These papers represent key schistosomiasis endemic regions. Overall, Lyne et al address a very important area of work that still remains highly understudied and this merits future work. Having said this, they have a preprint Lyne, B., Besong, M. E., Nabatte, B., Tinkitina, B., Oryema, J. B., Kabatereine, N. B., Lewington, S., & Chami, G. F. “Co-occurrence of alcohol use and Schistosoma mansoni infection: prevalence, patterns, and risk factors in rural Uganda.” medRxiv preprint (2025). Can this not be referenced as unpublished but can shed more light on general conclusions for this paper?

*Response:*

We thank the reviewer for this comment and appreciate them noting our related work. This preprint was made available after the search, screening, and synthesis stages of this scoping review were completed. We agree that the findings of this preprint are relevant to this research area, and have emphasised in the Discussion (Paragraph 10) that there is a need for additional empirical work to strengthen our conclusions.

Comment 9

- In populations studied- were they long term exposed/chronic or recent infections and what impact did this have on infection vs cirrhosis?

*Response:*

We thank the reviewer for this important question. The majority of included studies assessed current infection status using microscopy or antigen-based diagnostics, which reflects current detectable infection rather than cumulative exposure. No studies explicitly assessed infection chronicity or history. We have clarified this limitation in the Discussion (paragraph 6) by noting that the predominance of binary, cross-sectional data prevents the distinction between recent and chronic infection and limits inference regarding progression to cirrhosis: “Additionally, the reliance on binary measures of current schistosome infection limits the ability to assess the impact of chronic and cumulative infection on outcomes such as cirrhosis”.

Comment 10

- Were there any differences in species studied on study outcomes?

*Response:*

We thank the reviewer for this question. The ability to assess species-specific differences in study outcomes is limited by the predominance of studies focusing on *S. mansoni* and by substantial heterogeneity in outcomes assessed across species. Only one study investigated infection with *S. haematobium*, which focused on bladder pathology, an outcome which was not considered in any of the other studies. Of the three studies on *S. japonicum* (Li et al., 2000), one did consider periportal fibrosis, an outcome which was also studied in the context of *S. mansoni*. We have clarified this in the Limitations subsection of the Discussion by adding: “Additionally, as the majority of the studies examined *S. mansoni*, there are not enough studies involving other schistosome species to allow for an assessment of species-specific effects on clinical outcomes.”

Comment 11

- Line 77- limited data on mortality rate of schistosomiasis unknown. This is not wholly true some estimates put it approximately at 11,792 deaths per year globally (WHO, 2023). However, this is estimation is due to hidden pathologies like liver and kidney failure, bladder cancer, and ectopic pregnancies related to female genital schistosomiasis.

*Response:*

We thank the reviewer for raising this point. Though, the WHO data is likely a gross underestimate of schistosomiasis-related mortality as it relies either on the Global Burden of Disease approximations or vital registry data that often is not reported by endemic countries. Regardless, we have added the following clarification: “Although schistosomiasis-related mortality is difficult to quantify and data is limited, recent World Health Organisation (WHO) estimates suggest approximately 11,792 deaths per year.” (Introduction, paragraph 1).

Comment 12

- Line 82: Reference (7) pertains specifically to a study specifically on HIV and does not address the co-occurrence of schistosomiasis and alcohol use in the fishing communities examined. This should be corrected. Line 84: Reference (8) represents a study specifically assessing liver cirrhosis and again there is no mention of occupational stresses of fishing promoting alcohol use as a coping mechanism. Again, correct this.

*Response:*

We thank the reviewer for noticing these issues with the referenced citations. We have revised the wording to more accurately reflect the cited literature, and the reference supporting alcohol use in fishing communities has been replaced with one that better aligns with the message we are conveying.

“Fishing communities represent settings where schistosomiasis risk and alcohol use are both prevalent (Kuteesa et al., 2019), as populations experience prolonged water contact, increasing exposure to schistosome infection, and high levels of alcohol use have been reported due to unique social and occupational contexts (Bonnevie et al., 2020).” (Introduction, paragraph 1).

References

Bonnevie, E., Kigozi, G., Kairania, R., Ssemanda, J. B., Nakyanjo, N., Ddaaki, W. G., Ssekyewa, C., & Wagman, J. A. (2020). Alcohol use in fishing communities and men’s willingness to participate in an alcohol, violence and HIV risk reduction intervention: qualitative findings from Rakai, Uganda. Culture, Health & Sexuality, 22(3), 275–291. <https://doi.org/10.1080/13691058.2019.1587002>

Kuteesa, M., Weiss, H. A., Cook, S., Seeley, J., Ssentongo, J. N., Kizindo, R., Ngonzi, P., Sewankambo, M., & Webb, E. L. (2019). Epidemiology of Alcohol Misuse and Illicit Drug Use Among Young People Aged 15-24 Years in Fishing Communities in Uganda. SSRN Electronic Journal. https://doi.org/10.2139/SSRN.3420402
