# Supplementary material for: Clinical outcomes associated with schistosome infection and alcohol use: A systematic scoping review
Source: PLoS Negl Trop Dis. 2026 May 27;20(5):e0014371. doi: 10.1371/journal.pntd.0014371 (PMC13215488; doi:10.1371/journal.pntd.0014371)
Supplement: Attachment — Submitted filename: Response to reviewers.docx [file pntd.0014371.s008.docx]

Response to reviewers for manuscript: “Clinical outcomes associated with schistosome infection and alcohol use: a systematic scoping review”.

REVIEWERS’ COMMENTS

Reviewer 3

No comments given

Reviewer 4

Comment 1

- The original and the revision was read with the discussion between reviewers. All the issues pointed out by the first reviewers were addressed and the manuscript was improved overall. I suggest to add a limitation of including only English articles which might have led to missing studies.

*Response:*

We thank the reviewer for taking the time to read the manuscript and for this helpful suggestion. To clarify, no language restrictions were applied at the searching or screening stages. Translations were sought for any article not in English and assessed against the eligibility criteria. Ultimately, none of the studies for which translations were sought met the inclusion criteria, which is why all the included studies are in English. Therefore, we have not added language as a limitation, as the review did not exclude studies on this basis.

Reviewer 5

Comment 1

- The Abstract (Methods) refers to this review as a “systematic review”. As it is not a “systematic review”, please correct that to “scoping review”. Please also add the word “frequently” to this sentence in the Results section: “The most frequently reported clinical outcomes were…”.

*Response:*

We thank the reviewer for noticing these grammatical errors. In the Methods section of the abstract, the review is now referred to as a “scoping review”, and the third sentence of the Results section now reads as: “The most frequently reported clinical outcomes were periportal fibrosis (42.9%, 9/21), and biomarkers (19%, 4/21), including serum iron markers.” (Abstract, lines 29 and 38).

Comment 2

- METHODS: Database search and selection criteria (p.6). Please include information about the platforms from which you accessed the databases. For example, did you search the Web of Science (All Databases), or the Web of Science Core Collection? Did you search Global Health via Ovid?

*Response:*

We thank the reviewer for pointing out where extra information could be added. To clarify, we have added the platforms to the “Database search and selection criteria” section: “A systematic search was performed on 3 March 2025 across five databases: PubMed (1946 – present), Embase (via Ovid) (1974 – present), Web of Science Core Collection (1964 – present), Global Index Medicus (1901 – present), and Global Health (via Ovid) (1973 – present).” (Methods, page 6, lines 132-134).

Comment 3

- Thank you for explaining the rationale behind the search strategy to the previous peer reviewer. Please clarify which fields in the databases you searched (in reference to the search strategies available in Supplementary File S2). For example, did you search All Fields for the PubMed search, as you did for the Ovid databases (.mp.), and did you search the TOPIC field in Web of Science? Why did you not truncate alcohol* to include papers about alcoholism or alcoholics? Do you think that affects the results? You risk missing papers, eg. 89 papers in PubMed for the search for ‘alcohol’ not ‘alcohol*’: <https://pubmed.ncbi.nlm.nih.gov/?term=%28%28alcohol*+AND+schistosom*%29%29+NOT+%28alcohol+AND+Schistosom*%29&sort=date>

*Response:*

We thank the reviewer for their comments and for providing an example PubMed search. In PubMed, we applied the search string in All Fields – the equivalent of the .mp. search used with Embase and Global Health. This has now been indicated in Supplementary file S1. In the Web of Science Core Collection, we confirm that the TOPIC field was used to search title, abstract, and author keywords.

The reviewer’s example search is helpful, and we ran this search string. After reviewing all the results, we did not identify any additional records that would be eligible for inclusion in our scoping review (articles had either already been included or had an ineligible schistosome infection diagnostic, e.g., schistosomiasis based on presence of fibrosis rather than a test for current infection). Based on this, we have updated the search string described in the methods section and supplementary file S2 to reflect the corrected, more comprehensive search string: (Schistosom* OR Bilharzia* OR snail* fever) AND (alcohol* OR drink*). (Methods, line 136).

Comment 4

- Table 2: please add the correct citation details for each reference in the table, eg. Kavishe et al, 2021 [25]. Please then be consistent in your use of the number citation style instead of using the Harvard citation style (p.19, the whole Associations of alcohol use and schistosome infection to disease outcomes section), and make sure all references are cited. For example, p.19 line 358 you do not cite the study by Andrade et al, but p.20 (line 366) you do cite the study by Marinho et al.

*Response:*

We thank the reviewer for noticing these referencing omissions. References for each of the included studies have been added to Table 2 in the Results section, and the referencing style has been updated in the “Associations of alcohol use and schistosome infection to disease outcomes” section of the Results to ensure consistency. Finally, references have been added to later sections of the Results each time one of the included studies is mentioned again (Results, lines 333-387).

Comment 5

- References (pp.28-9): Please provide more citation details for [7] and [14].

*Response:*

We thank the reviewer for noticing the referencing issues with references [7] and [14]. These have been updated in the references section to now read as:

[7] World Health Organisation, “Global status report on alcohol and health and treatment of substance use disorders,” Geneva, Switzerland., 2024. [Online]. Available: <https://www.who.int/publications/i/item/9789240096745>. Accessed 21 April 2026.

[14] S. Zakhari, “Overview: How Is Alcohol Metabolized by the Body?,” *Alcohol Res Health.,* vol. 29, no. 4, pp. 245-254, 2006. PMID: 17718403.

Comment 6

- Thank you for providing me with the opportunity to review this paper. I am peer reviewing this paper in my professional capacity as an information specialist, adding to the reports that others have already provided you with. There is a gap in the literature for an evidence synthesis study about schistosome infection and alcohol use, and this scoping review provides valuable insights about future directions for research. This is original research which is of importance to researchers and policy makers, as well as being of interest to researchers and practitioners outside the field.

*Response:*

We thank and appreciate the reviewer for taking the time to read our manuscript and for their helpful suggestions, which have greatly improved the manuscript.
